# Supplementary material for: Environmental context-dependent activation of dopamine neurons via putative amygdala-nigra pathway in macaques
Source: Nat Commun. 2023 Apr 21;14:2282. doi: 10.1038/s41467-023-37584-9 (PMC10121604; doi:10.1038/s41467-023-37584-9)
Supplement: Supplementary file 1 — Supplementary Information [file 41467_2023_37584_MOESM1_ESM.pdf]

Supplementary Information

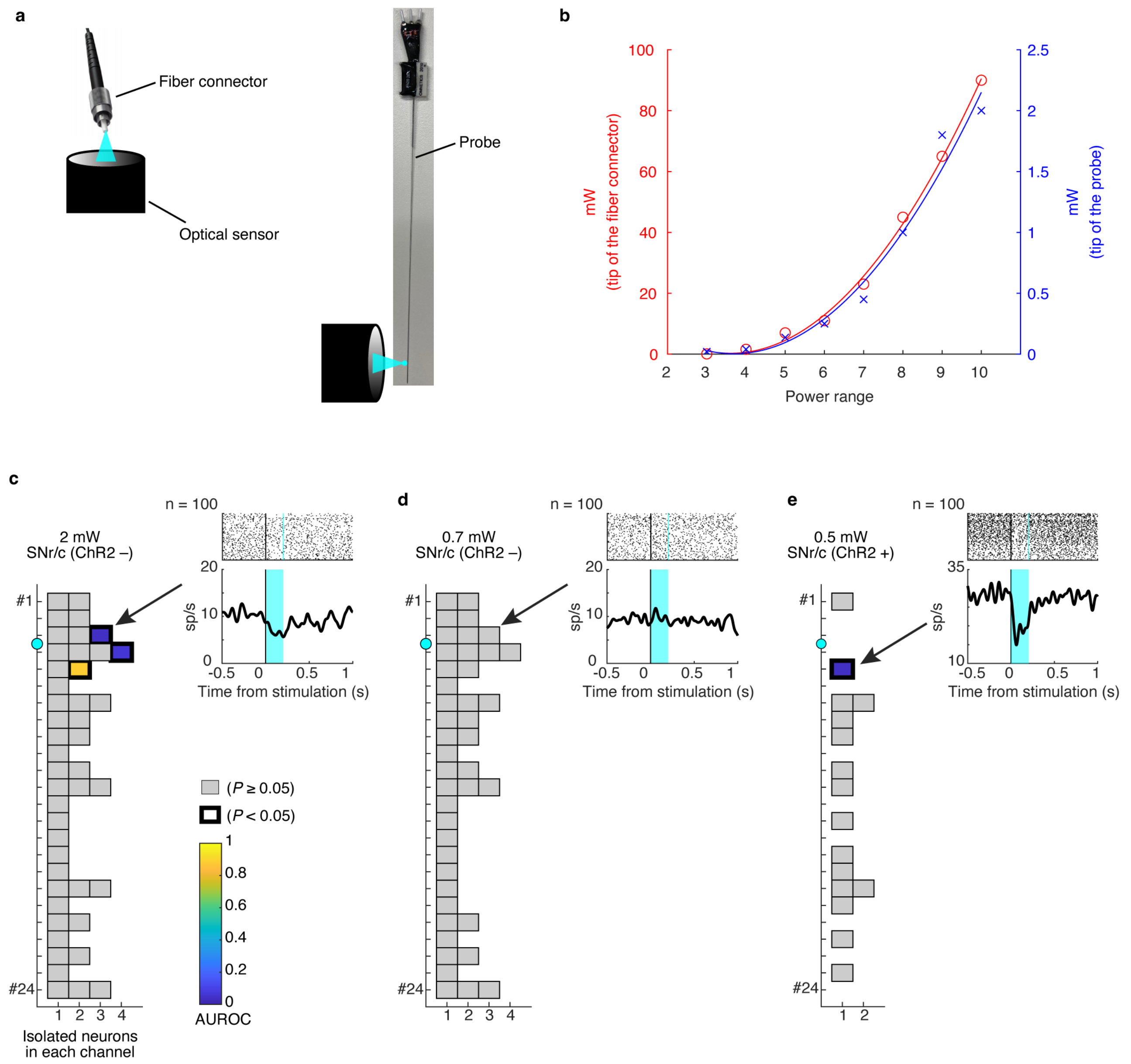

Fig. S1 Setting of stimulation intensity (related to Figure 2)

a, Two sites for measuring optical power (left: at the tip of the fiber connector, right: at the tip of the optrode).

b, Light intensities at the two places in different power rages. The ordinate shows measured light intensity (red: at the tip of the fiber connector, blue: at the tip of the optrode). The abscissa shows the power range on a scale of up to ten levels of the laser source.

c, **d**, Responses of substantia nigra neurons during applying optical stimulation to the neurons that are not expressed ChR2 (c: 2 mW, d: 0.7 mW intensity at the tip of the optrode). The ordinate shows the place of the recording contacts from top to bottom (#1 to #24). The abscissa shows the number of

isolated neurons in each contact. The place of the stimulation port of fiber optic is shown by the cyan dot (between #3 and #4 contacts). The color of each square shows the degree of modulation by the stimulation in each isolated neuron. The modulation score is defined as the area under the receiver operating characteristic (AUROC) based on the activity in stimulation trials vs. no-stimulation trials for each neuron. The scores were converted to the color scale if they were statistically modulated by stimulation ($P < 0.05$, two-sided paired t-test). The gray color indicates neurons that have no statistical significance. Example neurons were selected (black arrow) and the activities and raster plots are shown in the right panel. The cyan color in the panel shows the duration of the stimulation (200 ms). **e**, Responses of substantia nigra neurons during applying optical stimulation to the neurons that expressed ChR2 (0.5 mW).

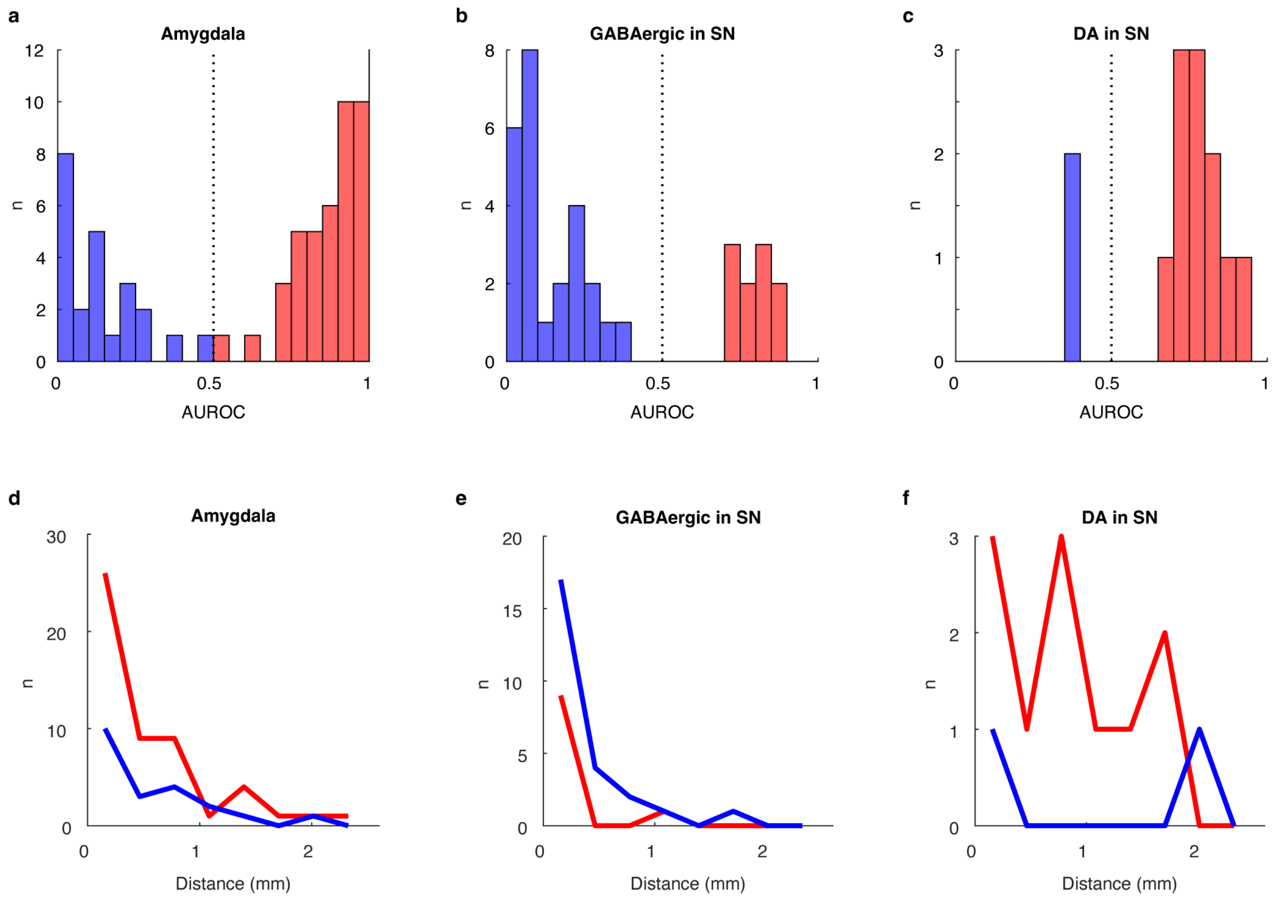

Fig. S2 Neuronal modulation and the effective range of the optogenetic stimulation (related to Figure 2)

a-c, Number of neurons that were modulated by the optogenetic stimulation ($P < 0.05$ t-test). AUROC >0.5 indicates the neuron that was excited by the stimulation and <0.5 indicates the neuron that was inhibited by the stimulation.

d-f, Distance of modulated neurons from the stimulation site.

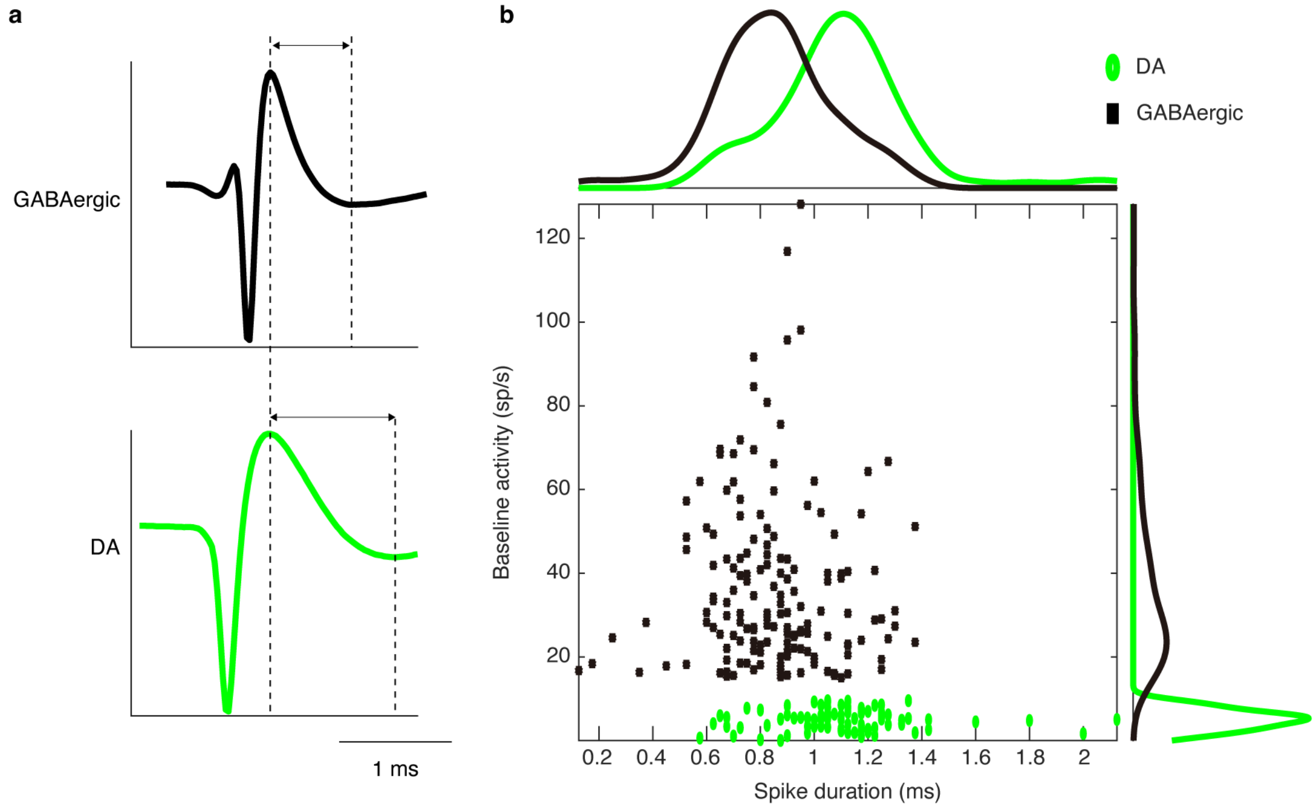

Fig. S3 Characteristic of putative GABAergic and putative DA neurons

a, Averaged spike shapes in each group.

b, The baseline firing rate (ordinate) and the spike duration (abscissa) for putative GABAergic and putative DA neurons. The spike duration was defined as the interval between voltage peak and subsequent trough (dotted line in a).

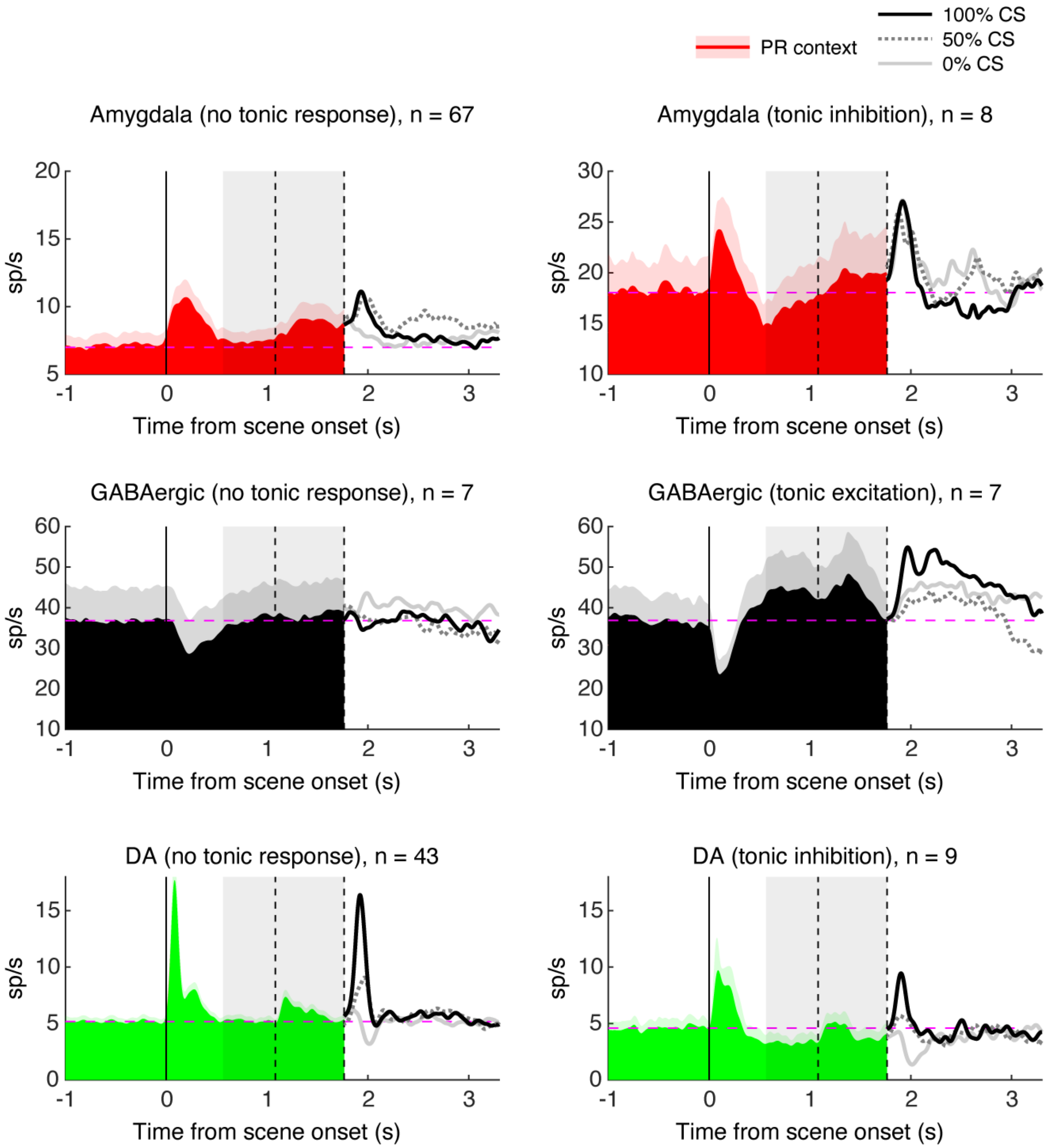

Fig. S4 All types of responses for environmental contexts (related to Figure 4)
Tonic excitation, tonic inhibition, and no tonic activity in amygdala, putative GABAergic, and putative DA neurons are shown. The format is the same as Figure 4a which shows the main responses during the task.

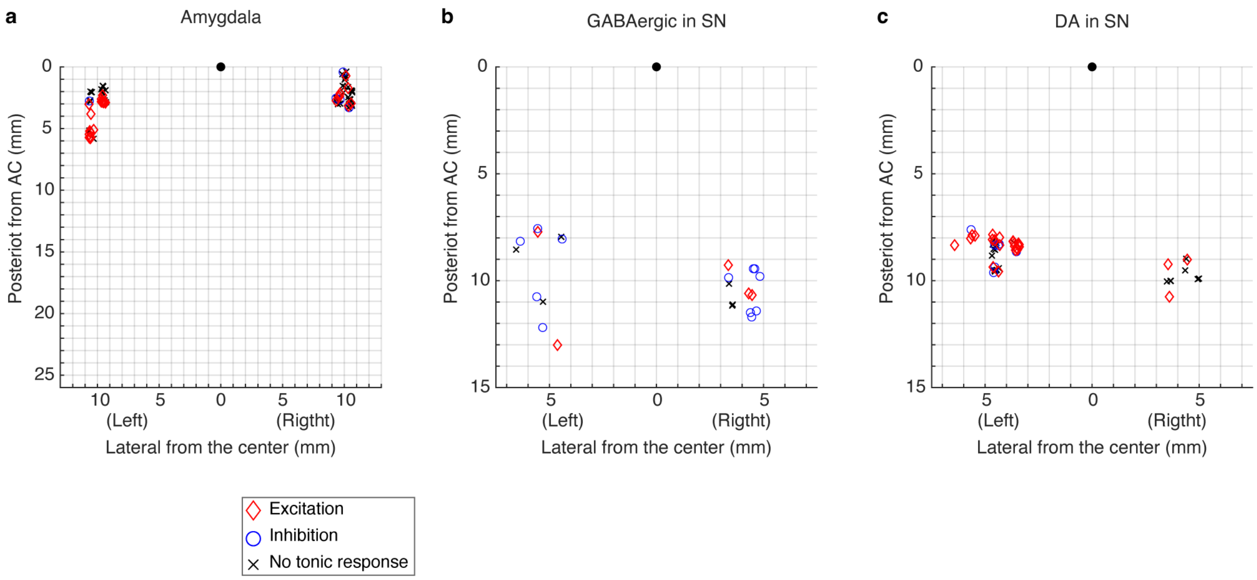

Fig. S5 AP level of the recording site

Recording locations of amygdala (a), putative GABAergic (b), or putative DA neurons (c) in Monkey SO. The ordinate indicates the distance from the center of the anterior commissure (AC) posteriorly. The abscissa indicates the distance from the center of AC laterally. Recording sites showing excited and inhibited responses to PR scene are indicated by red diamond and blue circle markers, respectively ($P < 0.05$, two-sided paired t-test). Cells with no significant response to PR are indicated by black markers.

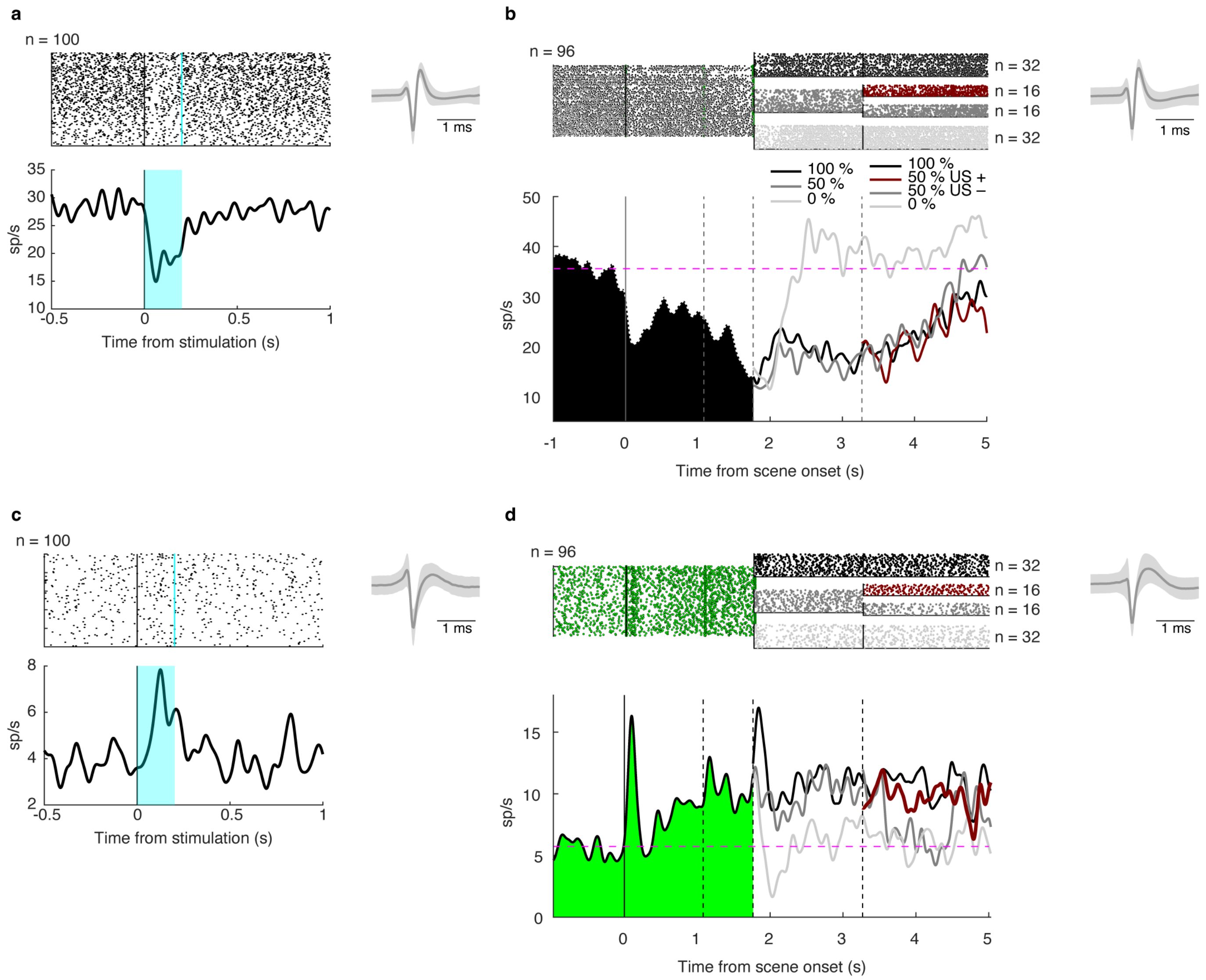

Fig. S6 Example neuronal responses (related to Figures 2 and 3)

a, Example putative GABAergic neuron that was inhibited by optogenetic stimulation of the amygdala's axon.

b, Responses to PR environment in the same putative GABAergic neuron. The format is the same as Figure 1.

c, Example putative DA neuron that was excited by optogenetic stimulation of the amygdala's axon. **d**, Responses to PR environment in the same putative DA neuron. The format is the same as Figure 1. The right panel in each figure shows averaged spike shape during each task. The shaded gray area indicates ± 1 SD.

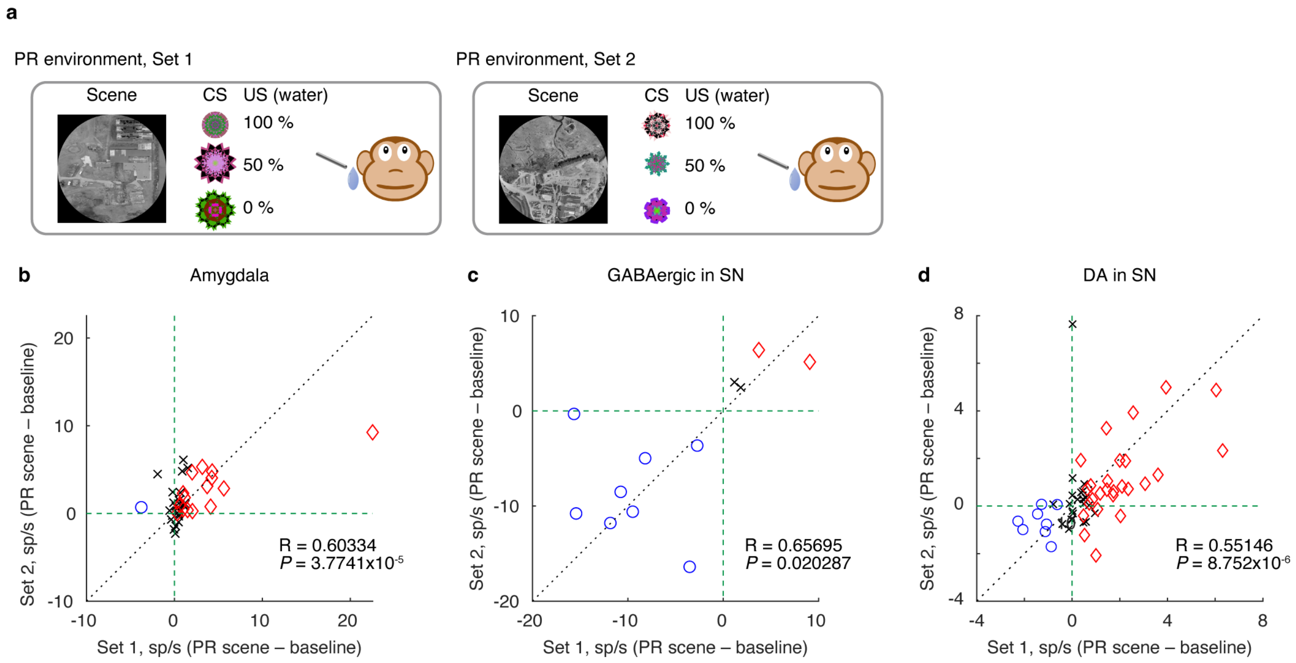

Fig. S7 Responses to different scene images

a, Replication of Pavlovian task with a second set of PR scene and fractal images.

b, Distribution of neuronal responses that were tested by both stimulus sets. The abscissa and ordinate show the response to the scene of set 1 or set 2, respectively, minus baseline activity. The red diamond, blue circle, or black cross indicate that the response increased, decreased, or no change respectively (P<0.05, two-sided paired t-test). The R and p-value are from Pearson's correlation.
