# Supplementary material for: Environmental context-dependent activation of dopamine neurons via putative amygdala-nigra pathway in macaques
Source: Nat Commun. 2023 Apr 21;14:2282. doi: 10.1038/s41467-023-37584-9 (PMC10121604; doi:10.1038/s41467-023-37584-9)
Supplement: Supplementary file 3 — Reporting Summary [file 41467_2023_37584_MOESM3_ESM.pdf]

nature portfolio

Corresponding author(s): Kazutaka Maeda

Last updated by author(s): Mar 10, 2023

Reporting Summary

Nature Portfolio wishes to improve the reproducibility of the work that we publish. This form provides structure for consistency and transparency in reporting. For further information on Nature Portfolio policies, see our Editorial Policies and the Editorial Policy Checklist.

Statistics

For all statistical analyses, confirm that the following items are present in the figure legend, table legend, main text, or Methods section.

n/a	Confirmed	
☐	☒	The exact sample size (*n*) for each experimental group/condition, given as a discrete number and unit of measurement
☐	☒	A statement on whether measurements were taken from distinct samples or whether the same sample was measured repeatedly
☐	☒	The statistical test(s) used AND whether they are one- or two-sided<br>*Only common tests should be described solely by name; describe more complex techniques in the Methods section.*
☒	☐	A description of all covariates tested
☐	☒	A description of any assumptions or corrections, such as tests of normality and adjustment for multiple comparisons
☐	☒	A full description of the statistical parameters including central tendency (e.g. means) or other basic estimates (e.g. regression coefficient) AND variation (e.g. standard deviation) or associated estimates of uncertainty (e.g. confidence intervals)
☐	☒	For null hypothesis testing, the test statistic (e.g. *F*, *t*, *r*) with confidence intervals, effect sizes, degrees of freedom and *P* value noted<br>*Give P values as exact values whenever suitable.*
☒	☐	For Bayesian analysis, information on the choice of priors and Markov chain Monte Carlo settings
☒	☐	For hierarchical and complex designs, identification of the appropriate level for tests and full reporting of outcomes
☐	☒	Estimates of effect sizes (e.g. Cohen's *d*, Pearson's *r*), indicating how they were calculated

Our web collection on statistics for biologists contains articles on many of the points above.

Software and code

Policy information about availability of computer code

Data collection	Behavioral tasks were controlled by a custom behavior-controlling system (Blip; available at http://www.robilis.com/blip/). Neuronal responses were recorded by OmniPlex Neural Recording Data Acquisition System (Plexon Inc).
Data analysis	Analysis was based on offline spike sorting using the Kilosort 2.5 algorithm followed by a manual curation in the Phy (Pachitariu et al., 2016). All statistical analyses were carried out using custom MATLAB scripts (MATLAB 2020b or 2022b).

For manuscripts utilizing custom algorithms or software that are central to the research but not yet described in published literature, software must be made available to editors and reviewers. We strongly encourage code deposition in a community repository (e.g. GitHub). See the Nature Portfolio guidelines for submitting code & software for further information.

Data

Policy information about availability of data

All manuscripts must include a data availability statement. This statement should provide the following information, where applicable:

- Accession codes, unique identifiers, or web links for publicly available datasets
- A description of any restrictions on data availability
- For clinical datasets or third party data, please ensure that the statement adheres to our policy

Data availability
Source data are provided in this manuscript and also deposited at https://figshare.com/s/4d73f51241b9c1358a62 (10.6084/m9.figshare.19105136).

Human research participants

Policy information about studies involving human research participants and Sex and Gender in Research.

Reporting on sex and gender	N/A
Population characteristics	N/A
Recruitment	N/A
Ethics oversight	N/A

Note that full information on the approval of the study protocol must also be provided in the manuscript.

Field-specific reporting

Please select the one below that is the best fit for your research. If you are not sure, read the appropriate sections before making your selection.

☒ Life sciences ☐ Behavioural & social sciences ☐ Ecological, evolutionary & environmental sciences

For a reference copy of the document with all sections, see nature.com/documents/nr-reporting-summary-flat.pdf

Life sciences study design

All studies must disclose on these points even when the disclosure is negative.

Sample size	The sample sizes are similar to those in the literature in the field, but no statistical methods were used to determine sample size. We recorded a total of 249 neurons across two brain areas (amygdala n=137, SN n=112) in two monkeys. These sample sizes are typical numbers for single unit recording studies; amygdala n=154 (Maeda, Kunimatsu, and Hikosaka, PLoS Biology, 2018), SNr n=109 (Amita et. al., Nature Communications 2020), DA in SNc n=103 (Matshumoto and Hikosaka, Nature 2009).
Data exclusions	All data with undefined spikes and irrelevant responses were excluded from data analyses.
Replication	All behavior experiments were repeated with the number of trials specified in the manuscript. Individual data points and standard error of the means were presented in the figures. All electrophysiological data was collected from the specified number of cells. Individual data points and standard error of the means were presented in the figures.
Randomization	All subjects and all stimulation/sham-stimulation timing were randomly assigned.
Blinding	For behavioral sessions with visual stimulation, experimenter knew the subject's group and applied corresponding visual stimulation. All other experimental sessions and their analyses were performed in blinded manner.

Reporting for specific materials, systems and methods

We require information from authors about some types of materials, experimental systems and methods used in many studies. Here, indicate whether each material, system or method listed is relevant to your study. If you are not sure if a list item applies to your research, read the appropriate section before selecting a response.

Materials & experimental systems

n/a	Involved in the study	
☒	☐	Antibodies
☒	☐	Eukaryotic cell lines
☒	☐	Palaeontology and archaeology
☐	☒	Animals and other organisms
☒	☐	Clinical data
☒	☐	Dual use research of concern

Methods

n/a	Involved in the study	
☒	☐	ChIP-seq
☒	☐	Flow cytometry
☒	☐	MRI-based neuroimaging

Animals and other research organisms

Policy information about studies involving animals; ARRIVE guidelines recommended for reporting animal research, and Sex and Gender in Research

Laboratory animals	We used two rhesus monkeys (Macaca mulatta) (monkey SO: 8.5 kg, 9y old, male, monkey BA: 9.0 kg, 8y old, male).
Wild animals	This study did not involve wild animals.
Reporting on sex	Sex was not considered in this design.
Field-collected samples	This study did not involve samples collected from the field.
Ethics oversight	All animal care and experimental procedures were approved by the National Eye Institute Animal Care and Use Committee (proposal number: NEI-622) and complied with the Public Health Service Policy on the Humane Care and Use of Laboratory Animals.

Note that full information on the approval of the study protocol must also be provided in the manuscript.
